# First report of an acute case of chagas disease in the municipality of Miraflores, Guaviare, Colombia

**DOI:** 10.17843/rpmesp.2024.412.13271

**Published:** 2024-06-13

**Authors:** José Ziadé Benítez, Diana Cedeño Díaz, Luz Alba Colorado, Laureano Mosquera Murillo, María Trinidad Orozco, Sandra Vallecilla, Julio Cesar Padilla, Mario J. Olivera

**Affiliations:** 1 Departmental Vector-Borne Diseases and Zoonosis Program, Guaviare Health Secretariat, Guaviare, Colombia. Departmental Vector-Borne Diseases and Zoonosis Program Guaviare Health Secretariat Guaviare Colombia; 2 Departmental Public Health Laboratory, Guaviare Health Secretariat, Guaviare, Colombia. Departmental Public Health Laboratory Guaviare Health Secretariat Guaviare Colombia; 3 Public Health Surveillance Area, Secretaría de Salud del Guaviare, Guaviare, Colombia. Public Health Surveillance Area Secretaría de Salud del Guaviare Guaviare Colombia; 4 Department of Entomology, Departmental Public Health Laboratory, Guaviare Health Secretariat, Guaviare, Colombia. Department of Entomology, Departmental Public Health Laboratory Guaviare Health Secretariat Guaviare Colombia; 5 Public Health Surveillance Area, Health Secretariat of Miraflores, Guaviare, Colombia. Public Health Surveillance Area SHealth Secretariat of Miraflores Guaviare Colombia; 6 Network for Knowledge Management, Research and Innovation in Malaria, Bogotá, Colombia. Network for Knowledge Management, Research and Innovation in Malaria Bogota Colombia; 7 Parasitology Group, National Institute of Health of Colombia, Bogota, Colombia. Parasitology Group National Institute of Health of Colombia Bogota Colombia

**Keywords:** Benznidazole, Case Reports, Chagas disease, Nifurtimox, Trypanosoma cruzi

## Abstract

We present a case of acute phase Chagas disease in a 40-year-old male patient from Vereda Buenos Aires, Municipality of Miraflores, Department of Guaviare. The patient attended the emergency department with fever, headache, asthenia, adynamia and dysuria. The blood smear and urinalysis were positive for symptomatic urinary tract infection, but negative for malaria. Five days later the diagnosis of acute phase Chagas disease was confirmed after a positive result for Trypanosoma cruzi. The patient was treated with nifurtimox and benznidazole, his contacts and risk areas were investigated, an active entomological community and institutional search was carried out, as well as in the reservoirs, finally, laboratory surveillance for possible cases of infection in the community was conducted. Five cases with similar symptoms were identified, but parasitological tests were negative. Health education measures were implemented to prevent the spread of the disease.

## INTRODUCTION

Chagas disease, caused by the parasite *Trypanosoma cruzi*, is transmitted mainly by the infected feces of hematophagous triatomine bugs, but can also be transmitted in a variety of ways, including blood transfusions, organ and tissue transplants, mother-to-child transmission, laboratory accidents, and oral ingestion ^1^^,^^2^. The incubation period varies according to the mode of transmission: 3 to 22 days for oral transmission, 4 to 15 days for vector-borne transmission, and 8 to 160 days for transfusions and transplants ^3^. The disease has an acute and chronic phase. Most patients are asymptomatic or experience nonspecific febrile symptoms in the acute phase ^(^^4^. The chronic phase is divided into two forms: indeterminate (latent) and determinate (clinical), which include cardiac, digestive and cardiodigestive manifestations ^5^.

The estimated global prevalence of infection is 6 to 8 million people, mainly in Latin America ^6^. Several departments in Colombia, including Santander, Cundinamarca, Boyacá, Casanare, Arauca and the Sierra Nevada de Santa Marta, have reported high prevalence rates ^7^. However, in the Department of Guaviare, although the *T. cruzi* antibody seroprevalence is 2.1%, most cases are imported from endemic areas, which suggests low circulation of the disease in the department. Therefore, the recent documentation of an acute case of Chagas disease in Guaviare is of great interest, as it represents the first evidence of autochthonous transmission in the region. This report serves as initial documentation of such a case in the department, highlighting the novelty and importance of this finding.

## CASE REPORT

**Figure 1:**
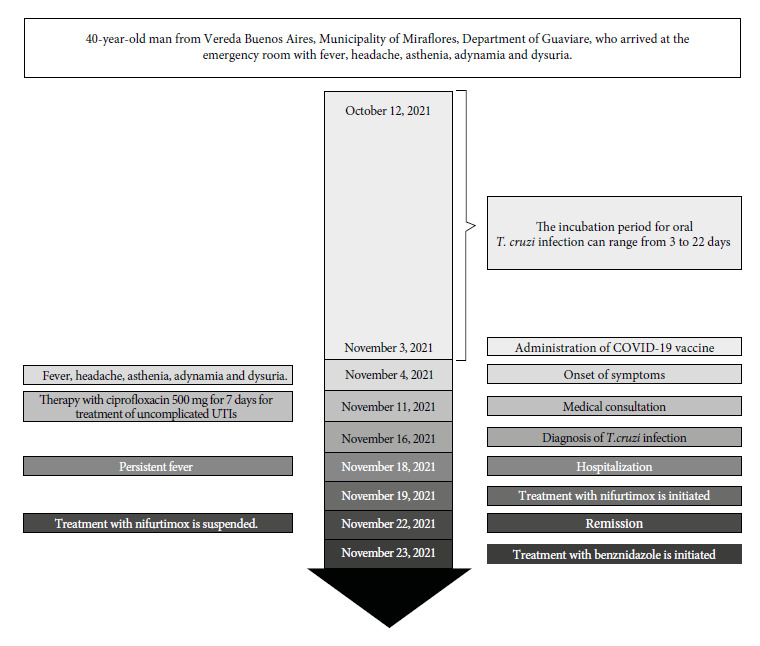
Complete timeline of acute Chagas disease, from incubation to hospitalization and initiation of treatment

**Figure 2.(A).T:**
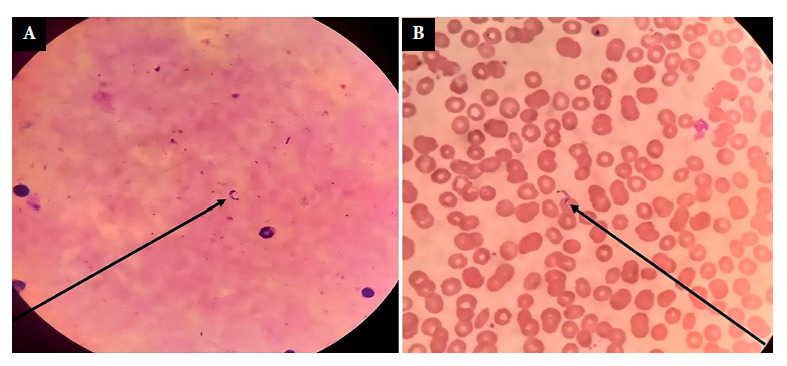
*cruzi* trypomastigotes in a thick blood smear from the patient, stained with Field’s stain at 100X. (B). *T. cruzi* trypomastigotes in a peripheral blood smear from the index case, stained with Wright at 100X.

This is the case of a 40-year-old male patient from Vereda Buenos Aires, Municipality of Miraflores, Department of Guaviare, who attended the emergency department of the Albert Schweitzer Hospital in Miraflores, level I, on November 11, 2021, with 7 days of unquantified fever, accompanied by headache, asthenia, adynamia, and dysuria (Figure 1). He had no photophobia, gastrointestinal or respiratory symptoms, and denied contact with persons positive for coronavirus disease (COVID-19). A thick blood smear test for malaria and a urinalysis were requested, the latter being positive for symptomatic urinary tract infection, which was later confirmed. Outpatient management began with ciprofloxacin 500 mg every 12 hours for 7 days and ibuprofen 400 mg every 8 hours for 5 days. After three days of treatment, the patient showed significant improvement of urinary symptoms. However, it is important to emphasize that the patient’s fever and general symptoms persisted. Five days later (November 16, 2021), a report was received indicating that the thick blood smear test was negative for malaria but positive for *Trypanosoma cruzi*, confirming the acute phase diagnosis of Chagas disease (Figure 2). Due to difficulties of access to the patient’s place of residence, he was informed and advised to go promptly to the nearest medical center (Albert Schweitzer Hospital in Miraflores, level I). The patient attended medical consultation on November 18, 2021 and was referred to Hospital San José del Guaviare, level II, for complementary tests and specialized management. The presence of parasites was confirmed again in the direct parasitological examination. The anti-T. cruzi IgG ELISA test using total extract antigens was positive. In addition, increased transaminases (aspartate transaminase 161.2 U/L; alanine transaminase 50.9 U/L) and increased C-reactive protein (24 mg/dL) were found. To date, the patient had had only occasional low-grade fevers with no other relevant symptoms or findings on physical examination (Table 1).

**Table 1:** Clinical features and laboratory findings in the follow-up of a case of acute Chagas disease, Colombia 2021.

Characteristics/date (dd/mm/yy)	18/11/21	19/11/21	20/11/21	21/11/21	22/11/21	23/11/21	24/11/21	25/11/21	26/11/21	27/11/21	28/11/21	30/11/21	04/12/21	05/12/21
WBC (mm^3^)	6900	-	-	-	13800	-	-	-	-	8000	-	-	10800	11200
LYM (%)	21	-	-	-	31.2	-	-	-	-	65.5	-	-	47.6	44.8
NEU (%)	52	-	-	-	60.3	-	-	-	-	25.5	-	-	40.8	42.5
EOS (%)	3	-	-	-	0.2	-	-	-	-	1.2	-	-	3.3	4.9
HGB (gm/dL)	12.3	-	-	-	12.4	-	-	-	-	11.3	-	-	12.5	12.8
PLA (/mm^3^)	245000	-	-	-	366000	-	-	-	-	309000	-	-	221000	247000
C-reactive protein (mg/dL)	24	-	-	-	-	-	-	-	-	-	-	-	-	-
Direct bilirubin (mg/dL)	0.62	0.12	-	-	0.17	-	-	-	-	0.13	-	-	0.12	-
Total bilirubin (mg/dL)	0.68	0.37	-	-	0.71	-	-	-	-	0.59	-	-	0.61	-
Glycemia (mg/dL)	138	-	-	-	105	-	-	-	-	-	-	-	-	-
ALT (IU/L)	161	125	-	-	72	-	-	-	-	127	156	117	55	-
PT Seg	12.4	12.4	-	-	11.9	-	-	-	-	11.4	-	-	-	-
PTT Seg	32.3	32.3	-	-	29.4	-	-	-	-	36.8	-	-	-	-
AST (IU/L)	50.9	48	-	-	107	-	-	-	-	111	107	49	28	-
BUN (mg/dL)	-	-	-	-	12.2	-	-	-	-	12.3	-	-	11.3	10.5
Creatinine (mg/dL)	-	-	-	-	0.71	-	-	-	-	0.92	-	-	0.85	0.8
Troponin (ng/mL)	Negative	-	-	-	-	-	-	-	-	-	-	-	-	-
Thick smear test	*T. cruzi* trypomastigotes	-	-	-	-	-	-	-	-	-	-	-	-	-
AKP (IU/L)	-	-	139	-	98	-	-	-	-	77	-	-	-	-
Albumin (g/dL)	-	-	3.2	-	3.9	-	-	-	-	3.6	-	-	-	-
Potassium (mmol/L)	-	3.8	-	-	-	-	-	-	-	-	-	-	-	4.5
Sodio (mEq/L)	-	137	-	-	135	-	-	-	-	-	-	-	-	137
CXR	-	-	-	-	Normal	-	-	-	-	-	-	-	-	-
ECG	Normal	-	-	-	Normal	-	-	-	-	-	-	-	-	-
ECO	-	-	-	-	LVEF 61%, Normal	-	-	-	-	-	-	-	-	-
EGD	-	-	-	-	Antral gastritis	-	-	-	-	-	-	-	-	-
Abdominal Ultrasound	-	-	-	-	-	Normal	-	-	-	-	-	-	-	-
Vital signs														
BT	37.5	36.6	36.5	36.1	36.9	36.2	36.5	37.1	36.7	37.3	36.8	37.4	36.6	36.2
HR	80	81	78	80	83	72	79	76	85	76	81	78	78	72
RR	18	18	19	20	20	18	17	18	19	17	21	19	22	19
BP	120/70	116/68	112/71	118/73	124/72	112/68	126/76	116/74	122/70	114/62	128/74	120/68	126/72	118/64

WBC: white blood cell count; LYM: lymphocytes; NEU: neutrophils; EOS: eosinophils; HGB: hemoglobin; PLA: platelets; ALT: alanine aminotransferase; PT: prothrombin time; PTT: partial thromboplastin time; AST: aspartate transaminase; BUN: blood urea nitrogen; AKP: alkaline phosphatase; CXR: chest X-ray; ECG: electrocardiography; ECHO: echocardiography; EGD: esophagogastroduodenoscopy; BT: body temperature in degrees Celsius (°C); HR: heart rate (beats per minute); RR: respiratory rate (breaths per minute); BP: blood pressure (mmHg); BMI: body mass index.

The patient started etiologic treatment with nifurtimox 120 mg (weight: 58 kg), the prescribed dose was 8 mg/kg/day, administered as one tablet every 8 hours. The treatment started on November 19, 2021. However, due to the likelihood of complications during the course of the disease and being the first clinical case reported in Guaviare, the responsible medical team decided to refer the patient to a more complex healthcare level. Thus, the patient was transferred to San Carlos level III Hospital, in the city of Bogota, on November 22, 2021, for evaluation and specialized management. At the time of admission, the patient still had fever and was on his fourth day of treatment with nifurtimox. Laboratory tests showed mild leukocytosis with neutrophilia, as well as elevated transaminases and circulating parasitemia (Table 1). No tests were performed to rule out the possibility of other diseases associated with a compromised immune system. In addition, the patient did not disclose any previous medical conditions or diseases. The responsible medical team decided to change the treatment to benznidazole 100 mg every 12 hours for 60 days because the dose of the previous medication was insufficient, and because benznidazole is the treatment of choice according to current clinical practice guidelines.

During hospitalization, the patient did not present hemodynamic alterations or changes, and tolerated the antitrypanosome treatment adequately without any adverse events. The cardiological tests were within normal limits. Finally, the patient was discharged in good health and continued supervised outpatient treatment at the Albert Schweitzer Hospital near his home. Subsequent follow-up showed no evidence of parasitaemia by direct parasitological methods, and anti-*T. cruzi* IgG serology was negative.

### Investigation of contacts and risk areas

The epidemiological field investigation showed that the patient identified as a native and resident of Miraflores, without a partner and with a six-year-old son. The patient frequently moved to two areas and moved infrequently to another: Vereda Mateguadua (zone 1), where he works and resides Monday through Friday; the center of the town of Buenos Aires (zone 2), where he visits his son, his only relative, on Saturdays and Sundays; and the town of Yavilla (zone 3), which he visits every 20 days, but does not stay overnight there. In addition, 16 direct contacts of the patient were identified, 5 in zone 1 and 11 in zone 2.

### Active community search

Probable cases of acute Chagas disease were identified during the active community search in zones 1 and 2 using the case definition recommended in the surveillance protocol. A total of 67 social and family contacts were evaluated, 5 of whom presented symptoms and underwent direct parasitological testing, while the other 62 asymptomatic contacts underwent serological testing. Both direct parasitological and serological tests were negative. In addition, malaria antigen and COVID-19 rapid tests were performed, the results of which were also negative. Finally, health education activities were carried out for the community.

### Active institutional search

An active institutional retrospective search was conducted at the Miraflores Hospital, in which the medical records of patients attended since October (one month before case presentation) were reviewed to find patients who met the case definition recommended in the surveillance protocol. As a result, 10 probable cases were identified and the serological tests were negative.

### Entomological investigation

Entomological surveys and detailed home inspections were carried out in zones 1 and 2 to identify risk areas related to the presence of the vector in bedrooms, mattresses, roofs and common areas. In addition, wild vector traps were installed around the house, but no vectors were found in them. On the other hand, a *Rhodnius prolixus* specimen was identified in a room of an educational institution located in zone 2; fresh examination was conducted on the specimen without finding intestinal contents.

### Reservoir investigation

Tomahawk traps were installed in zones 1 and 2 at strategic locations around houses with favorable biological conditions for the presence of wild animals. However, no reservoirs were captured in these traps. In addition, 10 blood samples were taken from canines, which were negative for *T. cruzi*.

## DISCUSSION

This report is the first clinical description of an autochthonous case of acute Chagas disease in the department of Guaviare. The case suggests possible oral transmission in an adult male who was incidentally detected and had an unusual clinical presentation. The patient received an accurate diagnosis and appropriate treatment, underscoring the importance of prompt and effective management of Chagas disease.

Possible oral transmission of *T. cruzi* should be suspected when acute symptoms manifest without bipalpebral edema or the presence of the Romaña sign ^8^. In addition, the absence of localized indurations, known as inoculation chagomas, commonly associated with vector-borne transmission, further strengthens the hypothesis of contamination through ingestion of infectious metacyclic forms ^8^.

This case is interesting because the patient lives in a non-endemic area, where other vector-borne diseases are prevalent ^9^. In addition, his initial diagnosis was incidental, as the first differential diagnosis considered the most common diseases in the region.

The medical team followed the current clinical practice guideline during the patient’s treatment and administered nifurtimox, which is one of the etiologic drugs available ^10^^-^^12^. However, at a more complex level of care to which he was referred, the treatment was changed to benznidazole, apparently following the guideline first-line regimen ^10^^,^^11^.

In our opinion, the patient was already receiving the appropriate etiologic treatment, albeit underdosed. Therefore, it was only necessary to adjust the dose to four tablets per day without the need to change the drug ^13^. Although the change in the current clinical practice guideline is justified, other important aspects had to be considered, such as the duration of treatment the patient had already been receiving, that it was the only option available at the screening site and that no adverse effects had occurred ^13^.

At post-treatment follow-up, both direct parasitological methods and anti-*T.cruzi* IgG serological testing were used to assess the effectiveness of treatment. Direct parasitological methods revealed no evidence of parasitemia, indicating successful elimination of *T. cruzi* from the patient’s bloodstream. Similarly, anti-*T. cruzi* IgG serological testing showed negative results, confirming the absence of antibodies to *T. cruzi*. This comprehensive evaluation provides strong evidence of parasite eradication, resulting in a positive outcome and suggesting a complete cure of *T. cruzi* infection ^1^^,^^2^^,^^14^^,^^15^.

It is important to note that, although not necessary, the patient was hospitalized for several days. Therefore, the recommended criteria for considering outpatient management with close surveillance should be properly applied in future cases ^16^. This measure can contribute significantly to health care professionals learning from the situation and improving patient-centered care ^17^.

One lesson learned is that management and prevention of future cases of Chagas disease in Guaviare will require a multifaceted approach. This approach should include surveillance of current cases, educational campaigns to raise awareness of the disease among the local population, and efforts to control vector populations ^16^. This measure can significantly contribute to health care professionals learning from the situation and improving patient-centered care ^17^.

In conclusion, our study shows that the population of the Department of Guaviare may be at risk for *T. cruzi* infection through oral transmission within the region. This highlights the importance for physicians to remain vigilant regarding acute Chagas disease as a possible zoonotic parasitic infection, especially in cases of prolonged febrile syndrome. Improved diagnostic prediction, management, and follow-up protocols are crucial to ensure optimal care for affected individuals.
