# Supplementary material for: First report of an acute case of chagas disease in the municipality of Miraflores, Guaviare, Colombia
Source: Rev Peru Med Exp Salud Publica. 2024 Jun 13;41(2):203–8. doi: 10.17843/rpmesp.2024.412.13271 (PMC11300699; doi:10.17843/rpmesp.2024.412.13271)
Supplement: Translation

# Translation: Primer caso agudo de enfermedad de Chagas en el municipio de Miraflores, Guaviare, Colombia

**Authors:** José Ziadé Benítez, Diana Cedeño Díaz, Luz Alba Colorado, Laureano Mosquera Murillo, María Trinidad Orozco, Sandra Vallecilla, Julio Cesar Padilla, Mario J. Olivera

**Affiliations:** 1 Programa Departamental Enfermedades Transmitidas por Vectores y Zoonosis, Secretaría de Salud del Guaviare, Guaviare, Colombia.; 2 Laboratorio Departamental de Salud Pública, Secretaría de Salud del Guaviare, Guaviare, Colombia.; 3 Área de Vigilancia en Salud Pública, Secretaría de Salud de Miraflores, Guaviare, Colombia.; 4 Red de Gestión de Conocimiento, Investigación e Innovación en Malaria, Bogotá, Colombia.; 5 Área de Vigilancia en Salud Pública, Secretaría de Salud de Miraflores, Guaviare, Colombia.; 6 Red de Gestión de Conocimiento, Investigación e Innovación en Malaria, Bogotá, Colombia.; 7 Grupo de Parasitología, Instituto Nacional de Salud de Colombia, Bogotá, Colombia.

**Keywords:** Benznidazol, Reportes de Caso, Enfermedad de Chagas, Nifurtimox, Tripanosoma cruzi

## Abstract

Se presenta un caso de enfermedad de Chagas en fase aguda en un paciente masculino de 40 años, procedente de la Vereda Buenos Aires, Municipio de Miraflores, Departamento del Guaviare. El paciente acudió a urgencias con fiebre, cefalea, astenia, adinamia y disuria. Se realizó un frotis de sangre y un análisis de orina, con resultados positivos para infección urinaria sintomática, pero negativos para malaria. Cinco días más tarde se confirmó el diagnóstico de enfermedad de Chagas en fase aguda tras recibir un resultado positivo para *Trypanosoma cruzi*. El paciente recibió tratamiento con nifurtimox y benznidazol, y se llevó a cabo una investigación de contactos y zonas de riesgo, búsqueda activa comunitaria e institucional, entomológica y de reservorios, y una vigilancia de laboratorio para detectar posibles casos de infección en la comunidad. Se identificaron cinco casos con síntomas similares, pero las pruebas parasitológicas fueron negativas. Se aplicaron medidas de educación sanitaria para prevenir la propagación de la enfermedad.

## INTRODUCCIÓN

La enfermedad de Chagas, causada por el parásito *Trypanosoma cruzi*, se transmite principalmente por las heces infectadas de los insectos triatominos hematófagos, pero también puede transmitirse de diversas formas, como transfusiones de sangre, trasplantes de órganos y tejidos, transmisión de madre a hijo, accidentes de laboratorio e ingestión oral ^1^^,^^2^. El período de incubación varía según la forma de transmisión: de 3 a 22 días para la transmisión oral, de 4 a 15 días para la transmisión por vectores y de 8 a 160 días para transfusiones y trasplantes ^3^. La enfermedad se presenta en una fase aguda y crónica. En la fase aguda, la mayoría de los pacientes son asintomáticos o experimentan síntomas febriles inespecíficos ^4^. La fase crónica se divide en dos formas: indeterminada (latente) y determinada (clínica), que incluyen manifestaciones cardíacas, digestivas y cardiodigestivas ^5^.

La prevalencia global estimada de la infección es de 6 a 8 millones de personas, principalmente en América Latina ^6^. En Colombia, varios departamentos, incluyendo Santander, Cundinamarca, Boyacá, Casanare, Arauca y la Sierra Nevada de Santa Marta, han reportado prevalencias altas ^7^. Sin embargo, en el Departamento de Guaviare, aunque se ha detectado una seroprevalencia de anticuerpos contra *T. cruzi* del 2,1%, la mayoría de los casos son importados de áreas endémicas, lo que sugiere una baja circulación de la enfermedad en el departamento. Por lo tanto, la reciente documentación de un caso agudo de enfermedad de Chagas en Guaviare es de gran interés, ya que representa la primera evidencia de transmisión autóctona en la región. Este informe sirve como documentación inicial de dicho caso en el departamento, resaltando la novedad e importancia de este hallazgo.

## REPORTE DE CASO

Se trata de un paciente masculino de 40 años, de la Vereda Buenos Aires, Municipio de Miraflores, Departamento de Guaviare, que acudió a urgencias del Hospital Albert Schweitzer en Miraflores, nivel I, el 11 de noviembre de 2021, con síntomas de fiebre no cuantificada de 7 días de duración, acompañado de dolor de cabeza, astenia, adinamia y disuria (Figura 1). No presentaba fotofobia, síntomas gastrointestinales ni respiratorios, y negó contacto con personas positivas para la enfermedad por coronavirus (COVID-19). Se solicitó una gota gruesa para malaria y un análisis de orina, siendo este último positivo para infección urinaria sintomática, lo cual se confirmó posteriormente. Se decidió el manejo ambulatorio con ciprofloxacina 500 mg cada 12 horas durante 7 días e ibuprofeno 400 mg cada 8 horas durante 5 días. Después de tres días de tratamiento, el paciente mostró una mejoría significativa en los síntomas urinarios. Sin embargo, es importante enfatizar que la fiebre y los síntomas generales del paciente persistieron. Cinco días después (16 de noviembre de 2021), se recibió un informe que indicaba que la prueba de gota gruesa fue negativa para malaria pero positiva para *Trypanosoma cruzi*, confirmando el diagnóstico de fase aguda de la enfermedad de Chagas (Figura 2). Debido a las dificultades de acceso al lugar de residencia del paciente, se le informó y recomendó que acudiera rápidamente al centro médico más cercano (Hospital Albert Schweitzer en Miraflores, nivel I). El paciente asistió a la consulta el 18 de noviembre de 2021 y fue remitido al Hospital San José del Guaviare, nivel II, para exámenes complementarios y manejo especializado. En el examen parasitológico directo, se confirmó nuevamente la presencia de parásitos. La prueba de ELISA IgG anti *T. cruzi* utilizando antígenos de extractos totales mostró una reacción positiva. Además, se evidenció un aumento de las transaminasas (aspartato transaminasa 161,2 U/L; alanina transaminasa 50,9 U/L) y un aumento de la proteína C reactiva (24 mg/dL). Hasta la fecha, el paciente solo había tenido fiebres ocasionales de bajo grado sin otros síntomas o hallazgos relevantes en el examen físico (Tabla 1).

**Figura 1:** Línea de tiempo completa de la enfermedad de Chagas aguda desde el período de incubación hasta la hospitalización y el inicio del tratamiento.

**Figura 2:** (A). Tripomastigotes de *T. cruzi* en un frotis grueso de sangre del caso índice, teñidos con Field a 100X. (B). Tripomastigotes de *T. cruzi* en un frotis de sangre periférica del caso índice, teñidos con Wright a 100X.

**Tabla 1:** Características clínicas y resultados de laboratorio en el seguimiento de un caso de enfermedad de Chagas aguda, Colombia 2021.

Características/fecha	18/11/21	19/11/21	20/11/21	21/11/21	22/11/21	23/11/21	24/11/21	25/11/21	26/11/21	27/11/21	28/11/21	30/11/21	04/12/21	05/12/21
RGB (mm^3^)	6900	-	-	-	13800	-	-	-	-	8000	-	-	10800	11200
LYM (%)	21	-	-	-	31,2	-	-	-	-	65,5	-	-	47,6	44,8
NEU (%)	52	-	-	-	60,3	-	-	-	-	25,5	-	-	40,8	42,5
EOS (%)	3	-	-	-	0,2	-	-	-	-	1,2	-	-	3,3	4,9
HGB (gm/dL)	12,3	-	-	-	12,4	-	-	-	-	11.3	-	-	12,5	12,8
PLA (/mm^3^)	245000	-	-	-	366000	-	-	-	-	309000	-	-	221000	247000
Proteína C-reactiva (mg/dL)	24	-	-	-	-	-	-	-	-	-	-	-	-	-
Bilirubina directa (mg/dL)	0,62	0,12	-	-	0,17	-	-	-	-	0,13	-	-	0,12	-
Bilirubina total (mg/dL)	0,68	0,37	-	-	0,71	-	-	-	-	0,59	-	-	0,61	-
Glucemia (mg/dL)	138	-	-	-	105	-	-	-	-	-	-	-	-	-
ALT (IU/L)	161	125	-	-	72	-	-	-	-	127	156	117	55	-
PT Seg	12,4	12,4	-	-	11,9	-	-	-	-	11,4	-	-	-	-
PTT Seg	32,3	32,3	-	-	29,4	-	-	-	-	36,8	-	-	-	-
AST (IU/L)	50,9	48	-	-	107	-	-	-	-	111	107	49	28	-
BUN (mg/dL)	-	-	-	-	12,2	-	-	-	-	12,3	-	-	11,3	10,5
Creatinina (mg/dL)	-	-	-	-	0,71	-	-	-	-	0,92	-	-	0,85	0,8
Troponina (ng/mL)	Negativa	-	-	-	-	-	-	-	-	-	-	-	-	-
Gota gruesa	Tripomastigotes de *T. cruzi*	-	-	-	-	-	-	-	-	-	-	-	-	-
AKP (IU/L)	-	-	139	-	98	-	-	-	-	77	-	-	-	-
Albumina (g/dL)	-	-	3,2	-	3,9	-	-	-	-	3,6	-	-	-	-
Potasio (mmol/L)	-	3,8	-	-	-	-	-	-	-	-	-	-	-	4,5
Sodio (mEq/L)	-	137	-	-	135	-	-	-	-	-	-	-	-	137
CXR	-	-	-	-	Normal	-	-	-	-	-	-	-	-	-
ECG	Normal	-	-	-	Normal	-	-	-	-	-	-	-	-	-
ECO	-	-	-	-	LVEF 61%, Normal	-	-	-	-	-	-	-	-	-
EGD	-	-	-	-	Antral gastritis	-	-	-	-	-	-	-	-	-
Ultrasonido Abdominal	-	-	-	-	-	Normal	-	-	-	-	-	-	-	-
Signos vitales														
TC	37,5	36,6	36,5	36,1	36,9	36,2	36,5	37,1	36,7	37,3	36,8	37,4	36,6	36,2
FC	80	81	78	80	83	72	79	76	85	76	81	78	78	72
FR	18	18	19	20	20	18	17	18	19	17	21	19	22	19
PA	120/70	116/68	112/71	118/73	124/72	112/68	126/76	116/74	122/70	114/62	128/74	120/68	126/72	118/64

RGB: recuento de glóbulos blancos; LYM: linfocitos; NEU: neutrófilos; EOS: eosinófilos; HGB: hemoglobina; PLA: plaquetas; ALT: alanina transaminasa; PT: tiempo de protrombina; PTT: tiempo parcial de tromboplastina; AST: aspartato transaminasa; BUN: nitrógeno ureico en sangre; AKP: fosfatasa alcalina; CXR: radiografía de tórax; ECG: electrocardiografía; ECO: ecocardiografía; EGD: esofagogastroduodenoscopia; TC: temperatura corporal en grados centígrados (°C); FC: frecuencia cardiaca (latidos por minuto); FR: frecuencia respiratoria (respiraciones por minuto); PA: presión arterial (mmHg); IMC: índice de masa corporal.

El paciente inició el tratamiento etiológico con nifurtimox 120 mg, considerando su peso de 58 kg, la dosis prescrita fue de 8 mg/kg/día, administrada como una tableta cada 8 horas. El tratamiento comenzó el 19 de noviembre de 2021. Sin embargo, debido a la probabilidad de complicaciones en el curso de la enfermedad y siendo el primer caso clínico reportado en Guaviare, el equipo médico responsable decidió remitir al paciente a un nivel más complejo. Así, el paciente fue trasladado al Hospital San Carlos, nivel III de atención en la ciudad de Bogotá, el 22 de noviembre del 2021, para evaluación y manejo especializado. Al momento de su ingreso, el paciente aún presentaba fiebre y estaba en su cuarto día de tratamiento con nifurtimox. Los análisis de laboratorio indicaron una leve leucocitosis con neutrofilia, así como transaminasas elevadas y parasitemia circulante (Tabla 1). No se realizaron pruebas para descartar la posibilidad de otras enfermedades asociadas a un sistema inmunológico comprometido. Además, el paciente no reveló ninguna condición médica previa o enfermedades en su historial médico. Debido a que recibió una dosis insuficiente del tratamiento establecido y porque el benznidazol es el tratamiento de elección según las pautas actuales de práctica clínica, el equipo médico responsable decidió cambiar el régimen terapéutico establecido a benznidazol 100 mg cada 12 horas durante 60 días.

Durante la hospitalización, el paciente no presentó alteraciones hemodinámicas ni cambios en su cuadro clínico, y toleró adecuadamente el tratamiento *antitripanosoma* sin experimentar eventos adversos. Las pruebas cardiológicas realizadas estaban dentro de los límites normales. Finalmente, el paciente fue dado de alta en buen estado de salud y continuó el tratamiento ambulatorio supervisado en el Hospital Albert Schweitzer, cerca de su hogar. En el seguimiento posterior no se observó evidencia de parasitemia mediante métodos parasitológicos directos, y la serología anti-*T. cruzi* IgG resultaron negativos.

### Investigación de contactos y áreas de riesgo

Durante la investigación epidemiológica de campo, se identificó que el paciente es nativo y residente de Miraflores, sin pareja y con un hijo de seis años. Se detectaron dos áreas de movilización frecuente del paciente y una esporádica: Vereda Mateguadua (zona 1), donde trabaja y reside de lunes a viernes; el centro del pueblo de Buenos Aires (zona 2), donde visita a su hijo, su único familiar, los sábados y domingos; y el pueblo de Yavilla (zona 3), al que visita cada 20 días, pero no se queda a dormir en ese lugar. Además, se identificaron 16 contactos directos del paciente, 5 en la zona 1 y 11 en la zona 2.

### Búsqueda activa en la comunidad

Durante la búsqueda activa en la comunidad en las zonas 1 y 2, se identificaron casos probables de enfermedad de Chagas aguda utilizando la definición de caso recomendada en el protocolo de vigilancia. Se evaluaron un total de 67 contactos sociales y familiares, de los cuales 5 presentaron síntomas y se les realizaron pruebas parasitológicas directas, mientras que a los otros 62 contactos asintomáticos se les aplicaron pruebas serológicas. Tanto las pruebas parasitológicas directas como las serológicas arrojaron resultados negativos. Asimismo, se realizaron pruebas rápidas de antígenos de malaria y COVID-19, cuyos resultados también fueron negativos. Finalmente, se llevaron a cabo actividades de educación sanitaria para la comunidad.

### Búsqueda activa institucional

Se realizó una búsqueda activa institucional retrospectiva en el Hospital de Miraflores, en la cual se revisaron las historias clínicas de los pacientes atendidos desde octubre (un mes antes de la presentación del caso), con el objetivo de encontrar pacientes que cumplieran con la definición de caso recomendada en el protocolo de vigilancia. Como resultado, se identificaron 10 casos probables, a quienes se les tomaron muestras serológicas, cuyos resultados fueron negativos.

### Investigación entomológica

Se llevaron a cabo encuestas entomológicas e inspecciones detalladas de viviendas en las zonas 1 y 2 con el fin de identificar áreas de riesgo relacionadas con la presencia del vector en habitaciones, colchones, techos y áreas comunes. Además, se instalaron trampas para vectores silvestres alrededor de la casa, pero no se encontraron vectores en ellas. Por otro lado, en una institución educativa ubicada en la zona 2, se identificó un espécimen de *Rhodnius prolixus* en una habitación, para el cual se realizó un examen fresco sin encontrar contenido intestinal.

### Investigación de reservorios

Se instalaron trampas Tomahawk en las zonas 1 y 2 en puntos estratégicos alrededor de las casas que presentaban condiciones biológicas favorables para la presencia de animales silvestres. Sin embargo, no se capturaron reservorios en estas trampas. Además, se tomaron 10 muestras de sangre de caninos, las cuales resultaron negativas para *T. cruzi*.

## DISCUSIÓN

Este informe es la primera descripción clínica de un caso autóctono de enfermedad de Chagas aguda en el departamento de Guaviare. El caso sugiere una posible transmisión oral en un hombre adulto que fue detectado incidentalmente y tuvo una presentación clínica inusual. El paciente recibió un diagnóstico preciso y un tratamiento adecuado, subrayando la importancia de un manejo rápido y efectivo para la enfermedad de Chagas.

Se debe sospechar la posible transmisión oral de *T. cruzi* cuando los síntomas agudos se manifiestan sin edema bipalpebral o la presencia del signo de Romaña ^8^. Además, la ausencia de induraciones localizadas, conocidas como chagomas de inoculación, comúnmente asociadas con la transmisión por vectores, refuerza aún más la hipótesis de la contaminación a través de la ingestión de formas metacíclicas infecciosas ^8^.

Este caso es interesante porque el paciente proviene de un área no endémica, donde otras enfermedades transmitidas por vectores son prevalentes ^9^. Además, su diagnóstico inicial fue incidental, ya que el primer diagnóstico diferencial consideró las enfermedades más comunes en la región.

Durante el tratamiento del paciente, el equipo médico siguió la guía actual de práctica clínica y administró nifurtimox, que es uno de los fármacos etiológicos disponibles ^10^^-^^12^. Sin embargo, en un nivel de atención más complejo al que fue remitido, se decidió cambiar el medicamento que estaba recibiendo a benznidazol, aparentemente siguiendo el régimen de primera línea de la guía ^10^^,^^11^.

En nuestra opinión, el paciente ya estaba recibiendo el tratamiento etiológico adecuado, aunque subdosificado. Por lo tanto, solo era necesario ajustar la dosis a cuatro tabletas al día sin necesidad de cambiar el fármaco ^13^. Aunque el cambio en la guía actual de práctica clínica está justificado, otros aspectos importantes debieron ser considerados, como la duración del tratamiento que el paciente ya había estado recibiendo, que era la única opción disponible en el lugar de detección y que no se habían presentado efectos adversos ^13^.

En el seguimiento posterior al tratamiento, tanto los métodos parasitológicos directos como la prueba serológica anti-*T*.*cruzi* IgG se utilizaron para evaluar la efectividad del tratamiento. Los métodos parasitológicos directos no revelaron evidencia de parasitemia, lo que indica la eliminación exitosa del *T. cruzi* del torrente sanguíneo del paciente. De manera similar, la prueba serológica anti-*T. cruzi* IgG mostró resultados negativos, confirmando la ausencia de anticuerpos contra *T. cruzi*. Esta evaluación integral proporciona pruebas sólidas de erradicación del parásito, lo que resulta en un desenlace positivo y sugiere una cura completa de la infección por *T. cruzi*^1^^,^^2^^,^^14^^,^^15^.

Es importante destacar que, a pesar de no ser necesario, el paciente estuvo hospitalizado durante varios días. Por lo tanto, es esencial que, en el manejo de casos futuros, se apliquen adecuadamente los criterios recomendados para considerar el manejo ambulatorio con una vigilancia cercana ^16^. Esta medida puede contribuir significativamente a que los profesionales de la salud aprendan de la situación y mejoren la atención centrada en el paciente ^17^.

Una lección aprendida es que el manejo y la prevención de futuros casos de la enfermedad de Chagas en Guaviare requerirán un enfoque multifacético. Este enfoque debería incluir la vigilancia de los casos actuales, campañas educativas para concienciar sobre la enfermedad entre la población local y esfuerzos para controlar las poblaciones de vectores ^16^. Esta medida puede contribuir significativamente a que los profesionales de la salud aprendan de la situación y mejoren la atención centrada en el paciente ^18^.

En conclusión, nuestro estudio muestra que la población del Departamento de Guaviare puede estar en riesgo de infección por *T. cruzi* a través de la transmisión oral dentro de la región. Esto destaca la importancia de que los médicos permanezcan vigilantes con respecto a la enfermedad de Chagas aguda como una posible infección zoonótica parasitaria, especialmente en casos de síndrome febril prolongado. Los protocolos mejorados de predicción diagnóstica, manejo y seguimiento son cruciales para garantizar una atención óptima para las personas afectadas.
